# Characterization of *tmt-opsin2* in Medaka Fish Provides Insight Into the Interplay of Light and Temperature for Behavioral Regulation

**DOI:** 10.3389/fphys.2021.726941

**Published:** 2021-10-22

**Authors:** Theresa Zekoll, Monika Waldherr, Kristin Tessmar-Raible

**Affiliations:** ^1^Max Perutz Labs, University of Vienna, Vienna Biocenter, Vienna, Austria; ^2^Research Platform “Rhythms of Life, ” University of Vienna, Vienna BioCenter, Vienna, Austria

**Keywords:** behavior, non-visual opsins, temperature, medaka, deep learning algorithms, seasonality, light

## Abstract

One of the big challenges in the study of animal behavior is to combine molecular-level questions of functional genetics with meaningful combinations of environmental stimuli. Light and temperature are important external cues, influencing the behaviors of organisms. Thus, understanding the combined effect of light and temperature changes on wild-type vs. genetically modified animals is a first step to understand the role of individual genes in the ability of animals to cope with changing environments. Many behavioral traits can be extrapolated from behavioral tests performed from automated motion tracking combined with machine learning. Acquired datasets, typically complex and large, can be challenging for subsequent quantitative analyses. In this study, we investigate medaka behavior of *tmt-opsin2* mutants vs. corresponding wild-types under different light and temperature conditions using automated tracking combined with a convolutional neuronal network and a Hidden Markov model-based approach. The temperatures in this study can occur in summer vs. late spring/early autumn in the natural habitat of medaka fish. Under summer-like temperature, *tmt-opsin2* mutants did not exhibit changes in overall locomotion, consistent with previous observations. However, detailed analyses of fish position revealed that the *tmt-opsin2* mutants spent more time in central locations of the dish, possibly because of decreased anxiety. Furthermore, a clear difference in location and overall movement was obvious between the mutant and wild-types under colder conditions. These data indicate a role of *tmt-opsin2* in behavioral adjustment, at least in part possibly depending on the season.

## Introduction

Behaviors, besides physiology, are the main output of the nervous system. They are subject to extensive adjustments in response to internal and external environmental parameters. Behavioral adjustments or adaptations are vital and have evolved so that survival, even during adverse conditions or acute environmental changes, is improved. As many environmental conditions change rhythmically, so do behaviors (Häfker and Tessmar-Raible, 2020). Prominent examples are seasonal behaviors, including reproduction, hibernation, and migration (Gwinner, 1996; Dawson et al., 2001; Sharp, 2005; Klinner and Schmaljohann, 2020). For animals to accurately anticipate and prepare for an upcoming season, diverse environmental cues are being surveyed: light intensity and spectra, photoperiod, and temperature among others. The encounter with rather strong changes in these cues, for example very cold or warm temperatures, needs appropriate energy allotment and, hence, proper physiological and behavioral adaptations (Chen et al., 2020).

Medaka fish are teleosts, which, like humans, follow a diurnal activity rhythm and, in the wild, are subjected to intense seasonal changes with altering photoperiods and temperatures (ca. 4°C up to >30°C) (Hilgers and Schwarzer, 2019; Shintani et al., 2021). This species has a long-standing history as a research subject, enabling major scientific advancements, and has gained increased attention since its genome-sequencing project was completed, not only for developmental genetics but also for behavioral studies such as mating and social interactions (Kasahara et al., 2007; Kobayashi and Takeda, 2008; Kirchmaier et al., 2015; Okuyama et al., 2017). Additionally, medaka is a rather distant teleost fish species compared to zebrafish, presenting great value for comparative studies (Furutani-Seiki and Wittbrodt, 2004). The increasing number of inbred lines available and ease in maintaining and breeding these fish in a laboratory setting make them popular for various experimental studies (Leger et al., 2021). Especially, larval and juvenile stages are used for high throughput behavioral phenotyping, mostly in toxicological contexts (Hong and Zha, 2019). In the seasonal context, medaka are considered to be eurytherms, which are animals able to withstand temperatures on both cold and warm extremes (Shima and Mitani, 2004) and, therefore, must have evolved robust adaptation strategies. For instance, the carolina biological supply (CAB) strain arises from southern parts of Japan. This region is known as a subtropical and temperate zone, and photoperiod, as well as temperature, varies quite substantially depending on the season (Hirayama et al., 2006; Shintani et al., 2021). Light receptive molecules have started to emerge as important players in such seasonal behaviors (Shimmura et al., 2017; Nayak et al., 2020).

Many vertebrates use light-sensing proteins, called opsins, in order to seize and translate light information from their surroundings. Not only for vision but also non-visual purposes, such as the entrainment of internal circadian rhythms in mammals (e.g., reviewed in Foster and Kreitzman, 2014; Hang et al., 2016; Leung and Montell, 2017). Non-visual photoreception was already noted more than 100 years ago in experiments on blinded and pinealectomized minnows (von Frisch, 1911). A puzzling variety of opsins has been described, grouped according to their structural, functional, and temporal properties, and linked to non-visual/extraretinal photoreception in various vertebrate tissues, including the brain (Soni and Foster, 1997; Kojima and Fukada, 1999; Moutsaki et al., 2000; Philp et al., 2000a,b; Ekström and Meissl, 2003; Peirson et al., 2009; Porter et al., 2012; Foster and Kreitzman, 2014; Hunt et al., 2014; Davies et al., 2015). Pelagic fish, in particular, possess a whole array of non-visual opsins, e.g., ~25 *opsin* genes in Neoteleosts, to which the clade medaka fish belong, and even 42 *opsin* genes in Zebrafish, making them “swimming photoreceptors” (Davies et al., 2015; Beaudry et al., 2017). We have recently investigated the single and combined genetic removal of two non-visual opsins of the teleost multiple tissue (TMT) opsin group (Fontinha et al., 2021). In this study, we provide further detailed behavioral analyses of one of the two, *tmt-opsin2*, especially in the context of different light and temperature conditions, in order to start to obtain deeper insight into the interactions of different environmental parameters.

Teleost multiple tissue-opsins were suggested to function in tissue photoentrainment nearly 20 years ago (Moutsaki et al., 2003). They form together with the Encephalopsins a common family, named Encephalopsin and TMT-opsins = ETO Family (Fischer et al., 2013). TMT-opsins are subdivided again into three classes, TMT opsins 1, 2, and 3. Classes 1 and 2 are blue light-sensitive Gi/Go-coupled receptors, which have been found in known photoreceptive sites of the brain, as well as novel unexpected brain areas, like inter and motor neurons of the mid- and hindbrain (Fontinha et al., 2021; Sato et al., 2021). We have previously started to investigate the functional roles of *tmt-opsin1b* and *tmt-opsin2*, which share expression pattern characteristics (Fischer et al., 2013; Sato et al., 2021) and spectral sensitivity (Sakai et al., 2015). While *tmt-opsin1b* mutant fish exhibited behavioral phenotypes under blue light conditions, homozygous mutant *tmt-opsin2* alone did not (Fontinha et al., 2021). Given the rather complex phenotypes of double mutants at different developmental stages, we next wondered if a behavioral phenotype of *tmt-opsin2* mutants alone might become apparent under more complex and somewhat more naturalistic conditions.

Specific seasonal behaviors have already been observed in medaka fish, like decreased locomotion below a particular temperature threshold, altered color perception, as well as social interactions and winter-induced anxiety-like behaviors (Chen et al., 2012; Shimmura et al., 2018; Nakayama et al., 2020). With anxiety being a difficult trait to measure or assess, a recent publication has suggested the open-field test to be an appropriate tool for testing such a hypothesis by analyzing changes in thigmotaxis, in this context defined as the habit of avoiding central locations (Lucon-Xiccato et al., 2020).

Given the interlink between light and temperature as environmental co-variables over time, we wondered about the behavioral coping strategies of medaka under such combined conditions. Due to the recent implication of a member of the ETO group in light-dependent metabolic adaptations to temperature (Nayak et al., 2020), we aimed to test TMT-Opsin2 further for its potential in seasonal behavioral adaptation, mainly focusing on temperature changes. More specifically, do medaka juveniles, lacking *tmt-opsin2*, alter their locomotion under different, potentially seasonally relevant temperatures, like that shown for wild-types (Chen et al., 2012)? Can we detect specific behavioral states associated with either warm or cold conditions? For this purpose, we combined motion tracking with a deep learning algorithm in order to achieve a high throughput unbiased approach. A convolutional neuronal network (“loopy”) enabled us to acquire versatile and quantifiable data sets from video motion tracking and, in combination with scripting languages (Kil et al., 2020; Janisch et al., 2021; Mitoyen et al., 2021), to identify specialized behavioral phenotypes on a larger scale.

## Materials and Methods

### Fish Husbandry and Ethics Statement

All animal research and husbandry were conducted according to Austrian and European guidelines for animal research (fish maintenance and care approved under BMWFW-66.006/0012-WF/II/3b/2014, experiments approved under BMWFW-66.006/0003-WF/V/3b/2016, which is cross-checked by Geschäftsstelle der KommissionfürTierversuchsangelegenheitengemäß § 36 TVG 2012 p. A. Veterinärmedizinische Universität Wien, A-1210 Wien, Austria, before being issued by the BundesministeriumfürBildung, Wissenschaft und Forschung). Medaka fish (*O. latipes*) strains were kept in a constant recirculating system at ~26–28°C in a 16-h light/8-h dark cycle and bred using standard protocols (Murata et al., 2019). Collected embryos were kept at 28°C until hatching in an embryo rearing medium (ERM; 0.1% [w/v] NaCl, 0.003% [w/v] KCl, 0.004% [w/v] CaCl_2_ × 2 H_2_O, 0.016% [w/v] MgCl_2_ × 6 H_2_O, 0.017 mM HEPES, and 0.0001% [w/v] methylene blue). The Generation of the *tmt-opsin2* mutant line has been described previously (Fontinha et al., 2021).

### Recording Apparatus and Hardware Setup

We used an automated observation box called Kastl (http://loopbio.com/kastl/; Loopbio GmbH, Vienna, Austria). LED panels were provided by Marine Breeding Systems GmbH, St. Gallen, Switzerland. The observation box can tightly regulate the internal light and temperature environment.

### Behavioral Experiment Setup

**Figure 1:**
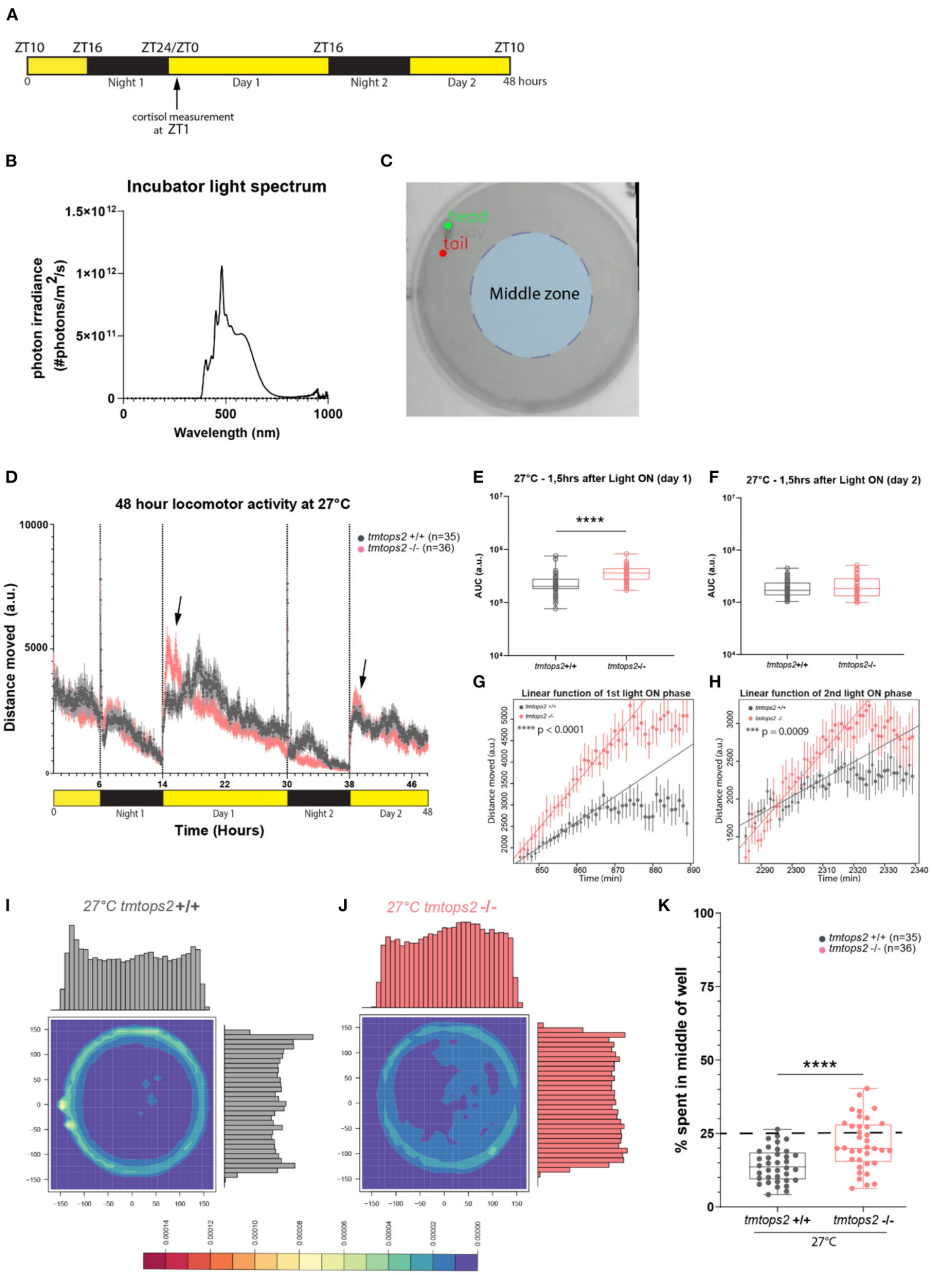
Experimental design and locomotor activity characterization at 27°C. **(A)** Schematic representation of the experimental design within the behavioral setup: 16-h light/8-h dark (L/D) regime. For cortisol measurements, larvae were sacrificed at ZT1 (arrow). **(B)** Light spectrum was used for all the experiments. **(C)** Schematic representation of the chosen middle zone (radius = 85 pixels) within the individual 6-well dish arena (radius = 170 pixels). **(D)** The average distance moved over the course of 48 h at 27°C. Each point represents the mean (± standard error of the mean, SEM) distance moved for the preceding 1 mi. Yellow and black boxes along the *x*-axis represent light conditions. Arrows indicate moments of increased mutant responses. **(E,F)** Locomotor activity (measured as the area under the curve, AUC, for 1.5 h) after **(E)** first and **(F)** second daytime periods. **(G,H)** Linear model fit to rise of activity after **(G)** first and **(H)** second light on from minimum to maximum represented as a line. Adjusted *R*-squared values are 0.9691 for **(G)** and 0.888 for **(H)**. Points represent the mean (± SEM) distance moved for the preceding minute. **(I,J)** Heat plots and histograms showing the density of the binned position data points. **(K)** Box plots showing the percentage of frames located in the middle zone; each dot represents one biological replicate, i.e., one juvenile larva. *n* = 35 (wt) and *n* = 36 (*tmtops2* mut), ****p ≤ 0.0001. a.u., arbitrary units; dashed line represents expected % if random. Data were collected from three experimental repetitions on different days, with up to 12 individual fish of each genotype/repeat. ***P ≤ 0.001.

Juveniles (ca. 21 days post fertilization (dpf); fed) were separated individually into 6-well plates filled with 12-ml fish system water. The plates were then placed into the Kastl and acclimatized to the internal conditions for 1 h prior to starting. A 48-h recording was performed with 15 frames per second. The light spectrum used can be seen in Figure 1B and the temperature was either set to 8 or 27°C.

### Data Analysis of Behavioral Experiments

Recorded videos were imported to the behavioral analysis interface Loopy (http://loopb.io, Loopbio GmbH, Vienna, Austria), where a neural network trained beforehand was used to accurately detect “head,” “body,” and “tail” of the juvenile fish. The coordinates of those key point detections for each frame were exported in csv format and used as input for further analysis with R [version 4.0.2 (2020-06-22)—“Taking Off Again”].

Distance moved over 48 h and the percentage of time spent in the middle area of the well were calculated from input files of decimated videos (3 frames per second). The area under the curve (AUC) and dark photokinesis were calculated and plotted as described in Fontinha et al. (2021) using GraphPad Prism 8. Visualization of the density of binned position data points was conducted using the function kde2d() from the R package MASS with normalized histograms on the margins.

The Hidden Markov model was fitted onto a training data set of 15 juvenile medaka fish over a time period of 16 h (to equally cover 8-h light and 8-h dark) and ensured the coverage of the temperature settings (27 and 8°C) used and genotypes (wild types and mutants) during the day and night periods. Specifically, three wild types and two mutants at 27°C and five wild types and five mutants at 8°C were used. The whole dataset was acquired under a long photoperiod of the 16-h light/8-h dark. The “head” key point was used as it was considered to most accurately show different behavioral states relevant to the whole fish, as fish are also strongly guided by the sensory input perceived and processed in the head/brain. The “head” point also has the advantage of showing several levels of movement while being more stable than the “tail” point. We reasoned that the latter could introduce higher levels of noise in the data. We used the prepData() and fitHMM() functions from the R package moveHMM (https://CRAN.R-project.org/package=moveHMM) (Michelot et al., 2016, 2019) and determined the starting parameters according to the guidelines given in the package vignettes, basically making an educated guess of initial means for each state from inspecting our data. Specifically, we assumed that a resting state would most likely have a small mean step length of around 0.1 px and a mean turning angle of around 0 rad with a high SD, while a highly active state would have a mean step length around 50 px and a mean turning angle of around 0 rad with a small SD. Step lengths were modeled by a gamma distribution while turning angles were modeled by von Mises distribution. The resulting model was evaluated by the cross comparison of state sequences with the actual video footage and comparison with a three-state model *via* Akaike Information Criterion (AIC) and then used to predict the sequence of states over 48 h for the different genotypes and temperature conditions. Decoding of the state sequences was performed using the viterbi () function. Details can be found in the provided R Scripts (refer to section Data Availability).

Linear regression models were fitted for the rise of activity after light on between minimum and maximum with the R function lm() from the package lsmeans; slopes were obtained with lstrends() for both genotypes.

### Cortisol Assay

The assay was performed on juvenile fish (21 dpf) as previously described (Fontinha et al., 2021). Larvae were sacrificed at ZT1 under a 16-h light/8-h dark light regime (Figure 1A). The concentration of cortisol was measured using an enzyme-linked immunosorbent assay (ELISA) kit (500360; Cayman Chemical, Ann Arbor, Michigan, USA).

### Statistical Analysis

The Kolmogorov–Smirnov test was performed to assess data distribution. When comparing normally distributed datasets, we performed a Student's *t*-test with Welch correction; otherwise, a Mann–Whitney test was conducted. The software used was GraphPad Prism 8, and a two-tailed *P*-value was always chosen: ^*^P ≤ 0.05; ^**^P ≤ 0.01; ^***^P ≤ 0.001; ^****^P ≤ 0.0001.

Both linear models (Figures 1G,H) were statistically significant with an overall *F*-statistic of 544.7 for the first light on and 183.3 for the second light on, with corresponding *p*-values of <2.2e−16, respectively. Pairwise comparison on the slopes was performed using the function pairs().

### Data Availability

All metadata and R scripts can be accessed *via* the following link: https://datadryad.org/stash/share/4KdmfgVzJ5ubIHRJqHDvLFCaqPpMojWpkZTTBGAhYaE.

## Results

### *tmt-opsin2* Mutant Fish Exhibit Increased Responses to Day Onset and Exhibit More Centralized Swimming Under Broad-Spectral Light and Summer Temperature Regime

In our initial analyses of *tmt-opsin2* mutants, we had not observed any effects on overall locomotion under a narrow wavelength band in the blue light range (Fontinha et al., 2021). Given the importance of light intensity and spectrum to opsin responses, we next wondered if changing the light conditions to a broader and more naturalistic spectrum (Figure 1B, Veedin Rajan et al., 2021) would lead to different results. We recorded juvenile *tmtops2* wild-type and homozygous mutant medaka fish and calculated the distance moved over time based on location data. The timeframe for this experiment was chosen to be comparable with our previous analyses under blue light conditions (Figure 1A).

At 27°C, the medaka juveniles, both the wild-types and *tmtops2* mutants, presented a clear diurnal activity pattern, as well as light-seeking behavior, known as dark photokinesis, upon night onset (Figure 1D; Supplementary Figure 1A). While we did not observe an overall locomotor phenotype in the mutant juveniles when quantifying the activity during the entire time span, except for night 2 [consistent with the results obtained under the previously used blue light conditions (Fontinha et al., 2021, Supplementary Figures 1B–E)], we observed that the juveniles tended to elevate their locomotion more in response to the start of the day than their wild-type counterparts (Figure 1D, arrows, Figures 1E,F). This was particularly obvious when calculating the slope of the responses by applying a linear model fit onto the data set (Figures 1G,H), but the difference vanished as daytime progressed (Figure 1D). We interpret from the stronger reaction that the *tmt-opsin2* mutation might make the juvenile fish more responsive to changes in environmental conditions, a feature that can likewise be observed under other conditions and their changes (as shown below). Additionally, we observed a temporal difference in the occurrence of an increase in swimming activity in the wild-type fish on day 1 relative to the mutant fish (and relative to the wild-type fish on day 2) (Figure 1D). This activity was observed in multiple larvae in three independent sets of experiments (individual distribution in each experiment following the order “shifted toward later times”/“no shift/”in-between”: experiment 1−8/3/1, experiment 2−5/4/2, and experiment 3−6/6/0). Given that this shift occurred in multiple fish from multiple independent experiments performed on different days, we consider this part of the normal wild-type behavior (and not an artificial “behavioral outlier”) that can, but does not have to, occur. As such a strong shift was not visible in the behaviors of wild-types in previous experiments (Fontinha et al., 2021), we hypothesize that external and/or internal factors modulate it, as those previous experiments were performed under different environmental light and temperature conditions. Furthermore, other factors that we did not precisely control, such as the density of fish during early development, might contribute to specific behavioral details. The understanding of such behavioral modulations and their underlying molecular and cellular mechanisms will be an informative tasks for the future.

We intentionally decided to perform our behavioral analyses in the relatively large 6-well dishes with a diameter of 3.5 cm, as compared with the often used smaller 12-, 24- or even 96-well-plates, to reduce behavioral artifacts (Wolter and Svoboda, 2020). This allowed us to observe that all the juveniles preferred to swim on the outskirts of the wells (Figure 1I; Supplementary Figures 2A,C). This preference of avoiding the middle of the well, known as thigmotaxis, was also present during the night (i.e., in darkness), albeit to a reduced extent (Supplementary Figures 2A vs. C, gray boxes Supplementary Figures 2I vs. J). This behavior has been used as a paradigm for anxiety-related behaviors (Simon et al., 1994; Godwin et al., 2012; Lucon-Xiccato et al., 2020). Given that it appeared to be relatively light-independent, we expected no effect of the *tmt-opsin2* mutation, even more since cortisol levels are not different between the wild types and *tmt-opsin2* mutants at 27°C (Supplementary Figures 3A,B). We quantified thigmotaxis by dividing the well into two zones, an outer zone and a middle zone (light blue shading in Figure 1C). We determined an area from the center with a radius half of the original well radius and calculated the percentage of frames falling into the middle area. The middle area accounts for ~25% of the overall area; hence, 25% of all movements should occur in this middle zone, if happening at random. The wild-type juvenile larvae are less in the middle area than expected at random (Figures 1I,K). Unexpectedly, the *tmt-opsin2* mutants avoided the middle portion of the well less, although the median still falls below the expected random (Figures 1J,K). This reduced thigmotaxis by the mutants could be observed and quantified during both the day and night periods (Supplementary Figures 2A,C vs. E,G,I,J gray vs. pink boxes). Heat plots with respective histograms for both *x* and *y* also provide an illustration for location preferences, with the wild-types peaking on the outsides of the well, while the mutants showed no clear peak but rather an even spread on both axes (Figures 1I,J; Supplementary Figures 2A,C vs. E,G). One aspect that should be considered in this context is the age-dependency of swimming behaviors in medaka, particularly the approach of open fields (Lucon-Xiccato et al., 2020). However, based on morphological criteria, time of hatching, as well as swimming behavior under blue light conditions (Fontinha et al., 2021), we see no evidence for a large developmental delay (for further details refer to Discussion). Thus, these data likely indicate that under more naturalistic white light conditions, *tmt-opsin2* modulates behavior, causing fish to respond faster to changes in light conditions and to be more explorative to open spaces, a feature that would likely have an impact on the species in the wild.

### Comparison of Warmer (Average Summer) vs. Acute Colder (Late Spring/Early Autumn) Temperature Conditions Reveals Temperature-Dependent and Independent Phenotypes of *tmt-opsin2*

**Figure 2:**
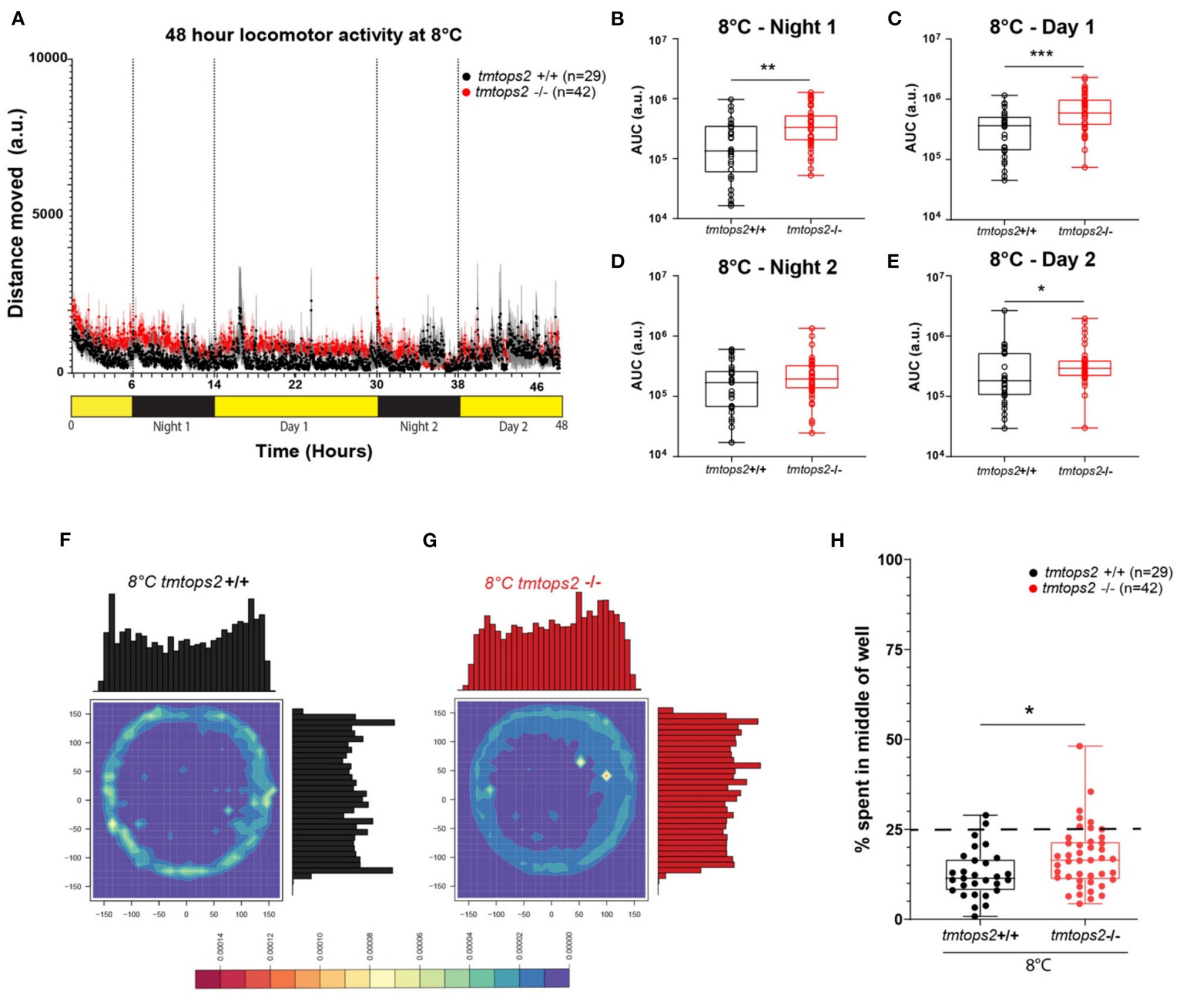
Locomotor activity characterization at 8°C. **(A)** Average distance moved over the course of 48 h at 27°C. Each point represents the mean (± SEM) distance moved for the preceding 1 min. Yellow and black boxes along the x-axis represent light conditions. **(B–E)** Locomotor activity (measured as AUC) during each separate night and day period. *n*= 29 (*tmtopsin2*-related wild type) and *n* = 42 (*tmtopsin2* homozygous mut). **(F,G)** Heat plots and histograms showing the density of the binned position data points. **(H)** Box plots showing the percentage of frames located in the middle zone; each dot represents one biological replicate, i.e., one juvenile larva. *n*= 29 (wt) and *n* = 43 (*tmtops2* mut). ***p ≤ 0.001, **p ≤ 0.01, *p ≤ 0.05. a.u.: arbitrary units; dashed line represents expected % if random. Data were collected from three to four experimental repetitions on different days, with up to 12 individual fish of each genotype/repeat.

Given that medaka locomotion is subject to seasonal adaptations (Chen et al., 2012; Shimmura et al., 2017; Nakayama et al., 2020), we next wondered how wild-type vs. *tmt-opsin2* mutant juveniles would handle acute changes to cooler temperature. When exposed to 8°C, the fish movement strongly decreased and diel changes were no longer distinguishable (Figure 2A). Photokinesis could still be observed when normalizing the datasets (Supplementary Figure 1F). In contrast to the situation observed at 27°C, the *tmt-opsin2* mutant juvenile larvae now moved overall significantly more than their wild-type counterparts at 8°C (Figures 2B–E), indicative of the temperature dependence of behavioral alteration. Further statistical analysis of the movement data of the different days by a two-way ANOVA further confirmed the differences between the wild-type and mutant fish (Supplementary Figure 5), revealing significant differences between the genotypes (Supplementary Figures 5A–C) and across time (Supplementary Figures 5B,D). While in the wild types only one-time point comparison was significant, the contrary was the case for mutant fish. For the mutant fish, all except one time point comparison was significantly different (Supplementary Figures 5B,D). This supports the notion that the reaction of *tmt-opsin2* mutant fish to changes in their environments is stronger, as it can also be seen for changes in light conditions at higher temperatures (see above, Figures 1G,H).

When analyzing the thigmotactic heat plots, we observed that, again, the mutants avoided the center spaces less, during day and night (Figures 2F–H; Supplementary Figures 2B,D vs. F,H–J: black vs. red boxes). Interestingly, while cortisol levels were slightly but significantly increased in the wild-types at 8°C, this was not the case for the *tmt-opsin2* mutants (Supplementary Figures 3A,B), further supporting the possible interpretation that mutations in *tmt-opsin2* cause reduced anxiety or stress in medaka fish, as also evident by the decreased avoidance of center areas by the mutants compared with the wild-types.

### A Hidden Markov Model Approach Unravels Specific Behavioral States of Juvenile Medaka Fish, Which Allows for the Detection of Specific *tmt-opsin2* Mutant Phenotypic Effects During Warm (Summer-Like) and Colder (Late Spring/Early Autumn-Like) Temperatures

**Figure 3:**
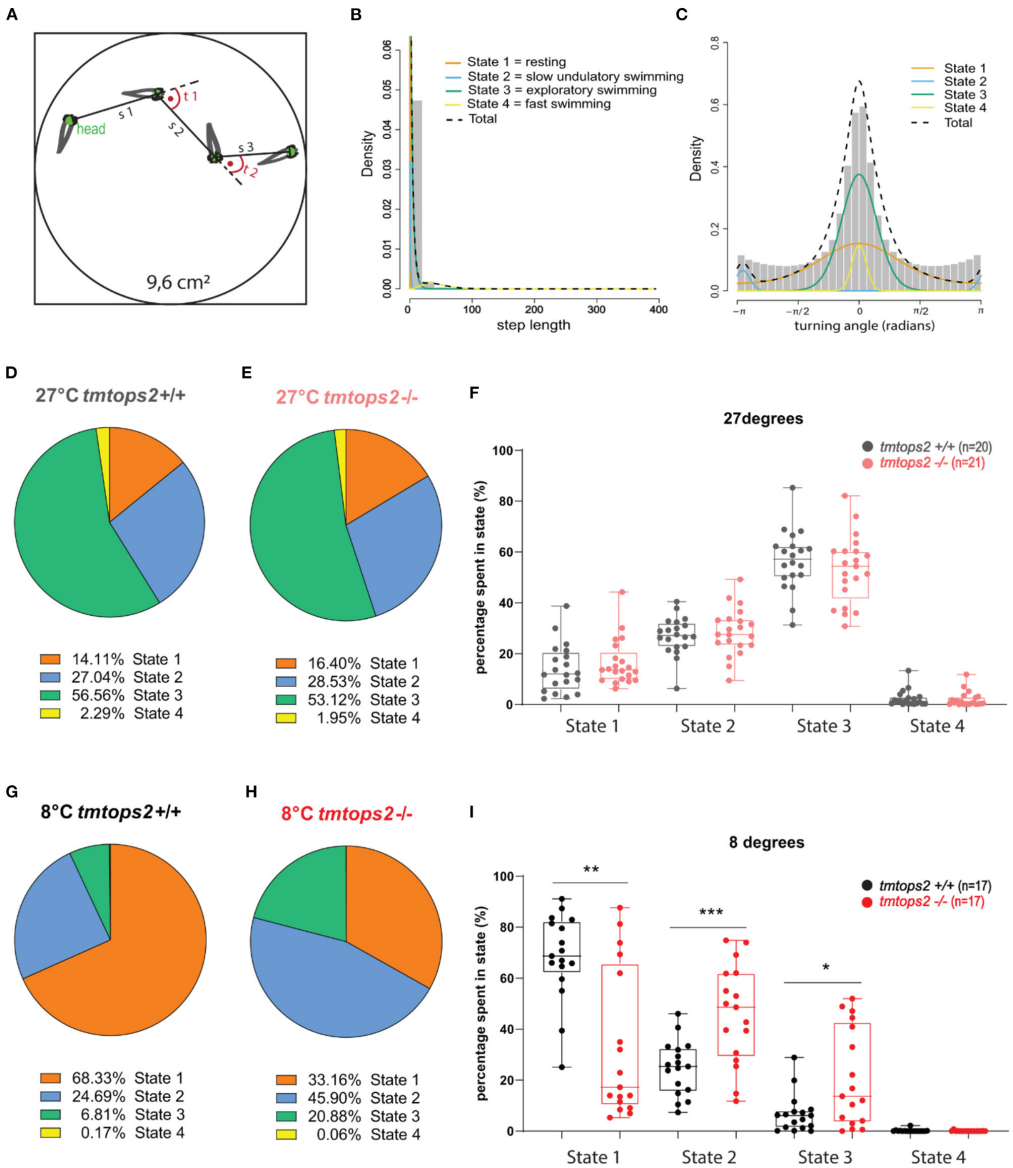
The combination of motion tracking and the Hidden Markov principle uncovers four different behavioral states. **(A)** Schematic representation of the Hidden Markov principle applied to our wells. s, step length; t, turning angle. **(B)** Histogram plotting step lengths and **(C)** Turning angles, overlaid with the individual curves representing distinct state distributions: orange, state 1/resting; blue, state2/slow undulatory movement; green, state 3/exploratory swimming; yellow, state 4/fast swimming. **(D,E,G,H)** Pie charts showing the ratios of each state in percentages. **(F,I)** State percentages from each biological replicate used, plotted individually per state and genotype. a.u., arbitrary units. ***p ≤ 0.001, **p ≤ 0.01, *p ≤ 0.05. *n* = 20/17 (wt 27/8°C) and *n* = 21/17 (*tmtops2* mut 27/8°C). For histograms of step length and turning angle of each state, as shown in Supplementary Figure 4.

While processing the video graphics datasets, we noticed temperature-dependent changes in juvenile *tmt-opsin2* mutant fish behavior that had not been covered by our quantitative analyses. Specifically, we noticed a distinct undulatory slow swimming style at colder temperatures (that in nature could mimic sudden cold nights in later spring or early autumn; Supplementary Movie 1), not observable at 8°C in the wild-type juveniles, which were rather immobile (Supplementary Movie 2). We, hence, aimed for a method that would be better suited to determine different behavioral “states.” Hidden Markov models have been used for sophisticated quantitative analyses on animal movement data (Woillez et al., 2016; Whoriskey et al., 2017; Bacheler et al., 2019; Conners et al., 2021). We employed the R package “moveHMM” on our data (Michelot et al., 2016, 2019). For model fitting, we used a tracking part of 16 h, including both the night and day periods, and ensured sufficient biological replicates of each temperature setting and genotype. For each frame-to-frame fish movement, a step length s and turning angle t were obtained, and these parameters were used as input data (Figure 3A). A four-state model was favored over a three-state one, after performing the model selection method (AIC). We then decided to name each state according to its step length and turning angle characteristics. State 1 was defined as resting, state 2 as very slow undulatory movement, state 3 as exploratory swimming, and finally state 4 as fast swimming (Figures 3B,C; Supplementary Figure 4). When we applied this model to our complete dataset, we observe no significant difference in behavioral state ratios at 27°C between the wild-types and *tmt-opsin2* mutants (Figures 3D–F; Supplementary Movie 3). Under colder conditions (mimicking acute temperature drops in summer or conditions that might arise in late spring/early autumn), though, the behavioral states of mutant juveniles differed significantly from those of their wild-type counterparts. While both genotypes never reached a long-lasting fast swimming pattern (state 4), the wild-types shifted toward a predominantly resting state, in contrast to the mutants that rather shifted to the undulatory swimming state 2 and rested ~30% less than the wild types (Figures 3G–I). Thus, the definition of behavioral states *via* an HMM approach successfully allowed for a more detailed description of general medaka behavior, and for the detection of more subtle behavioral differences present between the mutant and wild-type fish that depend on temperature.

### An Acute Switch to a Shorter Photoperiod Does Not Alter the Observed Locomotion Phenotypes

**Figure 4:**
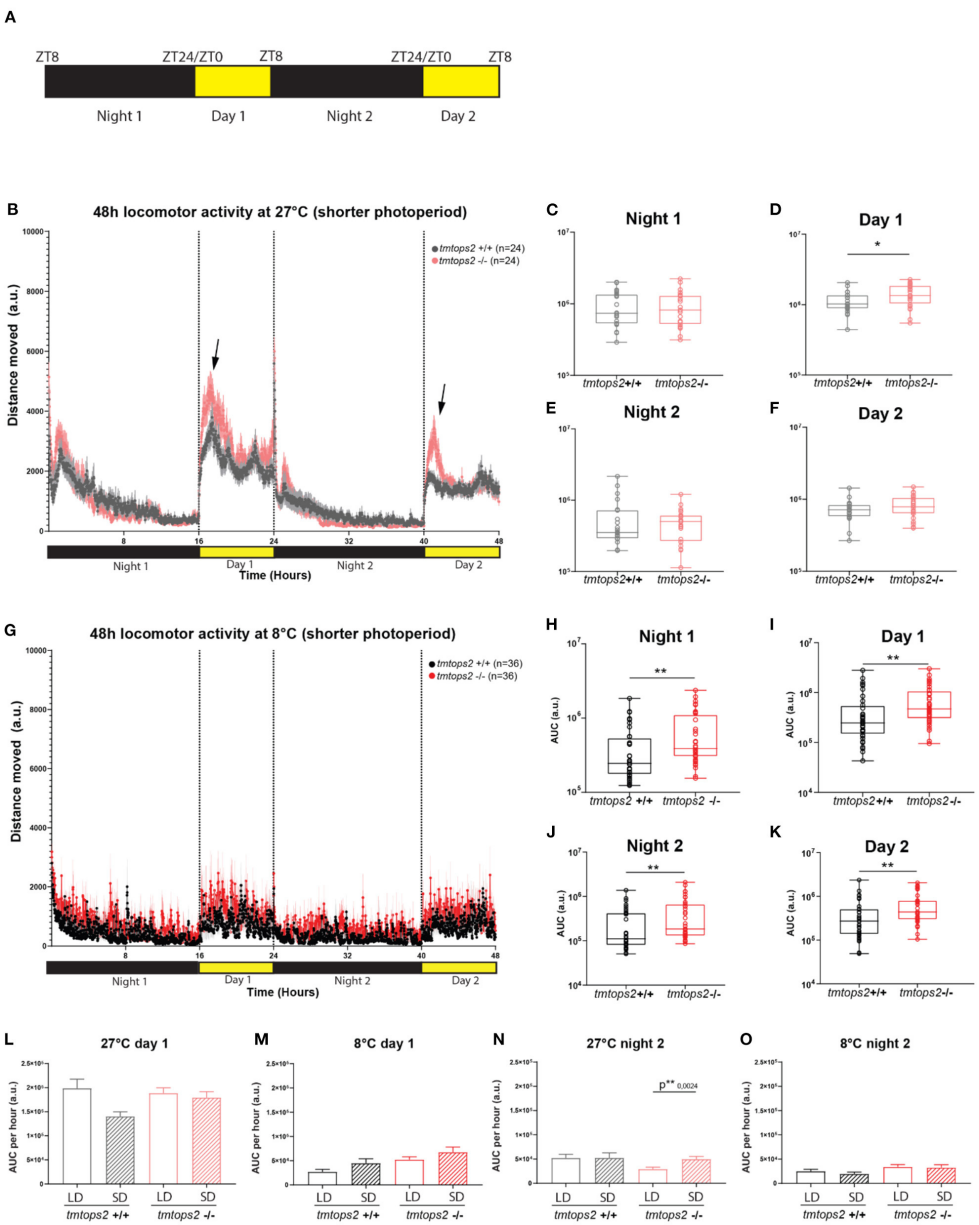
Acute temperature changes exhibit stronger effects than acute photoperiod changes. Distance moved quantified at 27 and 8°C with a short photoperiod of 8L/16D. **(A)** Schematic representation of the experimental design within the behavioral setup: 8 hours light/ 16 hours dark (L/D) regime. **(B)** The average distance moved over the course of 48 h at 27°C. Each point represents the mean (± SEM) distance moved for the preceding 1 min. Yellow and black boxes along the *x*-axis represent light conditions. Arrows indicate moments of increased mutant responses. **(C–F)** Locomotor activity (measured as AUC) during each separate night and day period at 27°C with a shorter photoperiod. **(G)** The average distance moved over the course of 48 h at 8°C. Each point represents the mean (± SEM) distance moved for the preceding 1 min. Yellow and black boxes along the *x*-axis represent light conditions. **(H–K)** Locomotor activity (measured as AUC) during each separate night and day period at 8°C with a shorter photoperiod. **(L–O)** Comparison of short photoperiod (short day, SD) vs. longer photoperiod (long day, LD) by measuring the AUC per hour for the first 8 h of day 1 at **(L)** 27 and **(M)** 8°C, and the first 8 h of night 2 for **(N)** 27 and **(O)** 8°C. *n* = 24/36 (27/8°C). a.u., arbitrary units. Bar plots: showing mean with (± SEM), **p ≤ 0.01, *p ≤ 0.05.

Given that it could be argued that the colder temperature with a long photoperiod is unnatural, we aimed to investigate how a similarly acute switch into a shorter photoperiod regime would affect the above observed phenotypes. We placed the wild-type and *tmt-opsin2* mutant juvenile medaka into the motion-tracking setup at 27 or 8°C under a photoperiod of 8-h light/16-h dark (Figure 4A). We observed that the switch to a shorter photoperiod while keeping the temperature at 27°C did not induce additional differences between the wild-type and mutant juveniles, compared with the data of the long photoperiod (Figures 1D, 4B). We still observe an increased activity upon day onset in the *tmt-opsin2* mutants compared with their wild-type relatives, but there was no overall difference in distance moved during night or day (Figure 4B arrows, Figures 4C–F). Similarly, when the juveniles were placed under both colder temperature and shorter photoperiod (conditions that might be relevant in late autumn/winter), the phenotype remained unchanged compared with the analyses performed under the long photoperiod (Figure 4G): the *tmt-opsin2* mutants moved significantly more throughout the entire experimental duration, as can be quantified with the area under the curve (AUC) (Figures 4H–K). An additional quantification of the AUC per hour for the first 8 h of day 1 and night 2, to directly compare movement at the most comparable time periods under long photoperiod (LD) and short photoperiod (SD), revealed only minor differences at 27°C and no difference at 8°C (Figures 4L–O). This suggests that acute changes in temperature cause stronger behavioral adjustments than acute changes in photoperiod, which makes ecological sense, given that acute changes in temperature can naturally occur, while those in photoperiod will only change slowly over a longer time period.

## Discussion

In this study, we provide the first insights into the impact of a broader naturalistic light spectrum and temperature changes on *tmt-opsin2* mutant juvenile Medaka. Our analysis strategy consisted of combining a motion tracker with a convolutional neuronal network, followed by downstream programming pipelines. This aided in the disentanglement of these complex and large datasets. We first acquired a baseline activity plot at 27°C and also assured the reliability of the tracking. Subsequently, we obtained comparable datasets at 8°C, which is a temperature that can acutely occur during spring/autumn seasons. The comparison of LD vs. SD photoperiods, allowed us to test for the interplay of different light and temperature conditions and their effects on non-visual opsin mutants vs. their wild-type relatives. We observed a significant decline in the activity of the juvenile larvae in the colder environment, a behavioral adaptation likely useful to quickly adjust energy expenditure and likely followed by metabolic changes to improve survival.

Interestingly, contrasting a previous study stating that medaka fish exhibit loss of phototaxis and light-seeking behavior (photokinesis) upon loss of illumination during winter conditions (Shimmura et al., 2017), we show here that the juveniles exhibited weak but present photokinesis at 8°C, even when the photoperiod was acutely significantly shortened (Figure 4F). The difference between the two studies might well be caused by different experimental procedures. The previous study examined adult medaka fish which had at least 2 weeks of acclimatization period, while we used juvenile larvae under acutely altered conditions. In this context, it is also noteworthy that in our results, behavioral differences between the two testing days can be observed (Figure 2A, comparison of wild-type first day/night with second day/night), indicative of possibly ongoing habituation. Both studies, thus, provide different insights into the plasticity of the fish to adjust their behavior to altered environmental conditions.

Before entering into the discussion of our manuscript, we want to emphasize that while we aimed to choose light and temperature conditions that could be interpreted in the context of relevant natural conditions, those experimental conditions are still laboratory conditions. In the future, they could certainly be further optimized to mimic specific environmental and natural aspects closer. This includes dusk and dawn or moonlight, as well as more gradual temperature changes, habituation times, and different combinations of temperature and light conditions. However, it should also be noted that no laboratory environment will fully mimic the natural environment, but laboratory conditions allow to specifically correlate behavioral and physiological responses to very defined environmental changes. From this perspective, our study provides relevant novel insight into responses to combined changes in light and temperature and the role of a non-visual opsin in mediating these responses.

“moveHMM” is a package available only relatively recently and enables a very unbiased and relatively easy to apply the method to establish and deduce distinct behavioral states from complex movement data (Michelot et al., 2016, 2019). This enabled us to gain a more specialized view of our locomotion data and show clear temperature-dependent behavior profiles: predominantly swimming and in motion at 27°C and more resting at 8°C. These two behavioral adaptations observed provide evidence that juvenile medaka induces a locomotion state of “hibernation” within an hour of exposure when subjected to colder temperatures. Reduced motor activity to the minimum occurs in many animals during naturally occurring cold periods, and the fact that this change happens rapidly in fish with no prior exposure hypothesizes an inborn coping mechanism. Altered locomotion now adds to our knowledge of behavioral adaption made by medaka under different temperature and light conditions. It, of course, needs to be acknowledged that we only tested one specific age. The responses at different ages might well be different (Lucon-Xiccato et al., 2020; Fontinha et al., 2021), an interesting aspect for future studies.

Early on, it was shown how temperature alone could alter reproductive behavior, and, recently, that temperature changes color perception *via* the *lws* opsin important for mate preference (Koger et al., 1999; Shimmura et al., 2017). The latter publication was the first to link a visual opsin with seasonal temperature adaptations in medaka fish. Our results in this study present the *tmt-opsin2* single mutant with a clear temperature-dependent phenotype and show it to respond differently, not only at the level of traveled distance but also when looking at their behavioral state ratios. This increased restlessness and undulatory movement is, however, still very slow when looking at the step length of the state (Supplementary Figure 4B). Of note, at 27°C, we already observe a different reaction to daylight. This difference in reaction suggests that losing *tmt-opsin2* changes only how the fish perceive the switch from darkness to daylight, as photokinesis at night onset was not altered (Supplementary Figures 1A,F). We could link neither the increased restlessness at 8°C nor the over-reaction to daylight at 27°C to an increase in stress levels, as only the wild-type juvenile larvae appeared to have elevated cortisol levels at 8°C (Supplementary Figures 3A,B).

We also assessed the thigmotactic traits of the juveniles, as the observation of tracking videos clearly showed the fish mainly swim on the edges of the wells. We were also able to extract this information from our tracking data, and confirm quantitatively that indeed the juvenile medaka fish did try to avoid open spaces. The notion of fearing the center or open space, called thigmotaxis, is a known concept in fish and was first reported in guppies (Mikheev and Andreev, 1993). In zebrafish, swimming more frequently toward or within the center has been associated with an anxiolytic effect (Maximino et al., 2010). This type of behavior has been linked to anxiety and has been used as a parameter for the effectiveness of drugs in pharmacological studies (Schnörr et al., 2012; Hong and Zha, 2019). Our results show that *tmt-opsin2* mutant juveniles spent significantly more time in the center, which is indicative of lower anxiety and stress levels. This is further confirmed by cortisol measurements when comparing wild-types and mutants between 8°C and 27°C. Nevertheless, further tests are required for a better understanding of the physiological and behavioral interconnection, given that decreased thigmotaxis in the *tmt-opsin2* mutants at 27°C was not correlated with lower cortisol levels. One additional explanation for the observed thigmotactic differences could be developmental delays in the mutants, as it has recently been shown that the time spent in the middle of a well is age-dependent in medaka (Lucon-Xiccato et al., 2020). However, as the published study found no differences between 10 and 30 days post hatching (approximately corresponding to 20 and 40 dpf) (Lucon-Xiccato et al., 2020), and we used ~21-dpf-old fish in our study with no evidence for developmental delay in *tmt-opsin2* mutant fish based on morphology, hatching, growth, or general swim behavior under blue light conditions (Fontinha et al., 2021), we think that developmental delay might not be a likely explanation. Of course, further studies will be required to further investigate this aspect, for instance, by further detailed analyses of neuronal connectivity and transmitter systems.

Evidence for the interplay between the impact of opsin photoreception and temperature is increasing. On the one hand, and as mentioned above, the visual *lws-opsin* has been shown to be regulated by seasonally relevant-temperature changes, impacting the execution of behaviors (Shimmura et al., 2017). Similarly, we observe that the role of the nonvisual opsin *tmt-opsin2* changes depending on temperature by affecting behavioral swim states, as well as the absence or presence of an increase overall locomotion at colder (as might be relevant in late spring/early autumn) vs. warmer (average summer-like) temperatures. This increased movement suggests in parallel ongoing metabolic changes, as increased movement levels will need to be accompanied by increased energy availability. Interestingly, another member of the ETO family, *encephalopsin* (*opn3*), has been linked to metabolic responses to temperature differences via a light-dependent impact on lipolysis in mammals (Nayak et al., 2020). While this effect appears to be a direct function of *encephalopsin* (*opn3*) in the fat tissue, non-visual opsin function in the brain can also be linked to light-dependent metabolic control, as has been shown for the neuropsin *opn 5*. This opsin, expressed in the preoptic area of the hypothalamus in mice and sensitive to deep violet/UVA light, has been associated with the suppression of thermogenesis in brown adipose tissue *via* its function in brain neurons (Zhang et al., 2020).

It remains for future studies to further disentangle the complex interplay of different opsins with each other, as well as other environmental parameters (such as temperature). Fish and other vertebrates have an amazing ability to modulate physiology and behavior to optimally adjust to environmental changes, the mechanisms behind them have just been started to be understood.

## Conclusion

In this study, we started to analyze the modulatory impact of the nonvisual opsin *tmt-opsin2* on behavioral modulation at different, naturally occurring, temperatures. The knockout of *tmt-opsin2* results in both temperature-dependent and independent altered behaviors. In a warm (average summer-like) temperature setting, the mutants respond to the abrupt onset of daylight differently than the wild-type. Under acute colder temperature settings (possibly relevant in late spring/early autumn), both wt and mutant fish decrease their motility, albeit *tmt-opsin2* mutants still maintained significantly higher movement levels. Additionally, irrespective of temperature, the mutant juveniles appear less stressed or anxious, as particularly evidenced by the increased exploration of open middle spaces (thigmotaxis). In addition to the more classical locomotion quantifications, we applied a hidden Markov model approach on our complex and relatively large behavioral datasets to distinguish distinct behavioral states. Four distinct states were observed within our data, and clear ratio differences were detectable for the two temperature conditions. Our approach revealed the role of *tmt-opsin2* in adapting the behavior of the fish to a colder environment. The exact link between this opsin and temperature needs further investigation but might hint toward a seasonally linked function, detecting colder conditions and as such preparing the animal for the upcoming season.

## Data Availability Statement

The datasets presented in this study can be found in online repositories. The names of the repository/repositories and accession number(s) can be found below: https://datadryad.org/stash, https://datadryad.org/stash/share/4KdmfgVzJ5ubIHRJqHDvLFCaqPpMojWpkZTTBGAhYaE.

## Ethics Statement

All animal research and husbandry were conducted according to Austrian and European guidelines for animal research (fish maintenance and care approved under BMWFW-66.006/0012-WF/II/3b/2014, experiments approved under BMWFW-66.006/0003-WF/V/3b/2016, which is cross-checked by Geschäftsstelle der Kommission für Tierversuchsangelegenheiten gemäß § 36 TVG 2012 p. A. Veterinärmedizinische Universität Wien, A-1210 Wien, Austria, before being issued by the Bundesministerium für Bildung, Wissenschaft und Forschung).

## Author Contributions

TZ performed the experiments together with MW, and analyzed the data. TZ and KT-R designed the study and interpreted the data. TZ, KT-R, and MW wrote the manuscript. All authors contributed to the article and approved the submitted version.

## Funding

This study was supported by the research platform Rhythms of Life of the University of Vienna and an FWF (http://www.fwf.ac.at/) SFB grant (#SFB F78), an FWF research project grant (#P28970), and funding from the European Research Council under the European Community's Horizon 2020 Programme ERC Grant Agreement 819952 to KT-R. TZ was supported by a uni:docs fellowship of the University of Vienna.

## Conflict of Interest

The authors declare that the research was conducted in the absence of any commercial or financial relationships that could be construed as a potential conflict of interest.

## Publisher's Note

All claims expressed in this article are solely those of the authors and do not necessarily represent those of their affiliated organizations, or those of the publisher, the editors and the reviewers. Any product that may be evaluated in this article, or claim that may be made by its manufacturer, is not guaranteed or endorsed by the publisher.
