# Supplementary material for: Characterization of tmt-opsin2 in Medaka Fish Provides Insight Into the Interplay of Light and Temperature for Behavioral Regulation
Source: Front Physiol. 2021 Oct 22;12:726941. doi: 10.3389/fphys.2021.726941 (PMC8569850; doi:10.3389/fphys.2021.726941)
Supplement: Supplementary file 4 [file Data_Sheet_1.pdf]

Supplementary Figures and Figure captions

Characterization of *tmt-opsin2* in medaka fish provides insight into the interplay of light and temperature for behavioral regulation

Theresa Zekoll[1,2], Monika Waldherr[1,2], Kristin Tessmar-Raible[1,2@]

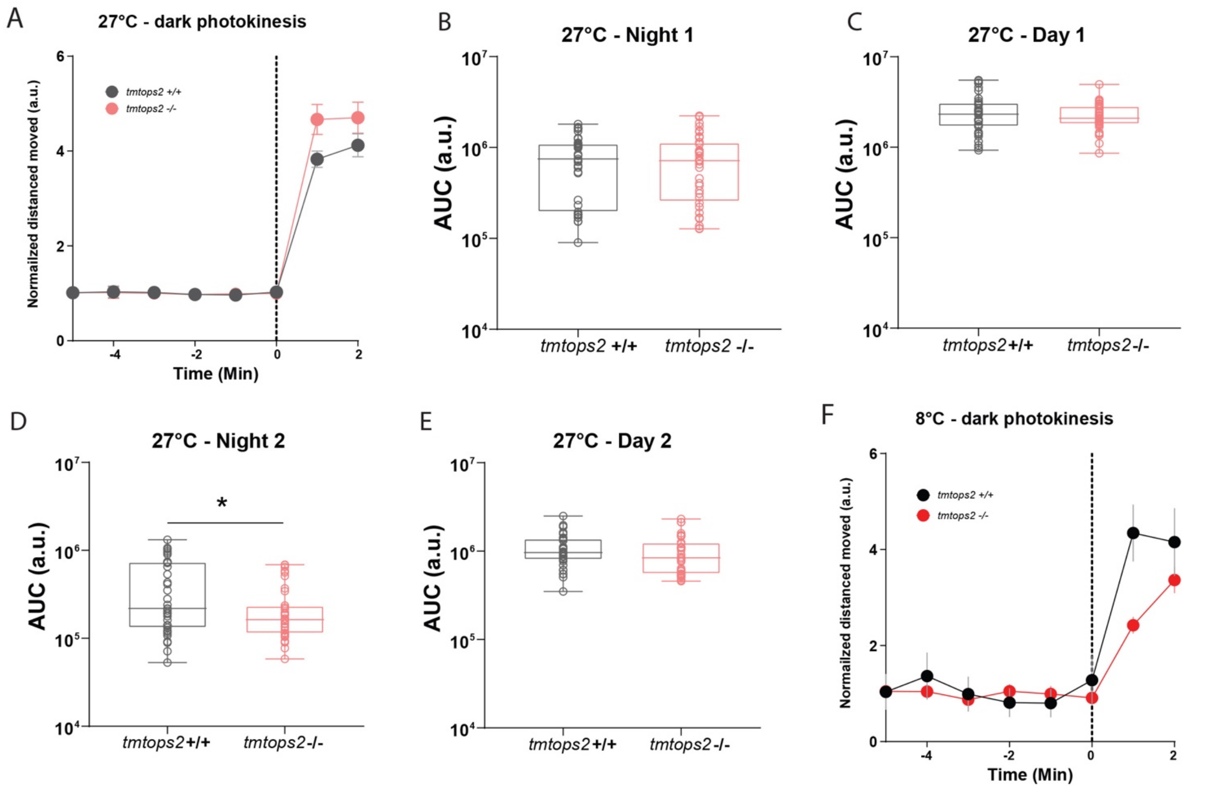

<u>Supplementary Figure 1:</u> refers to Figures 1D, 2A. (A) Dark photokinesis response of juveniles at 27°C, normalized to the average of the 5 min preceding darkness, each point represents the mean (±s.e.m.) normalized distance moved for 1min (time preceding). (B-E) Locomotor activity (measured as AUC) during each separate night and day period at 27°C. **** $p \leq 0.0001$, * $p \leq 0.05$.; a.u.: arbitrary units. n = 35/36. (F) Dark photokinesis response of juveniles at 8°C normalized to the average of the 5 minutes preceding darkness, each point represents the mean (±s.e.m.) normalized distance moved for 1min (preceeding), n = 29/42.

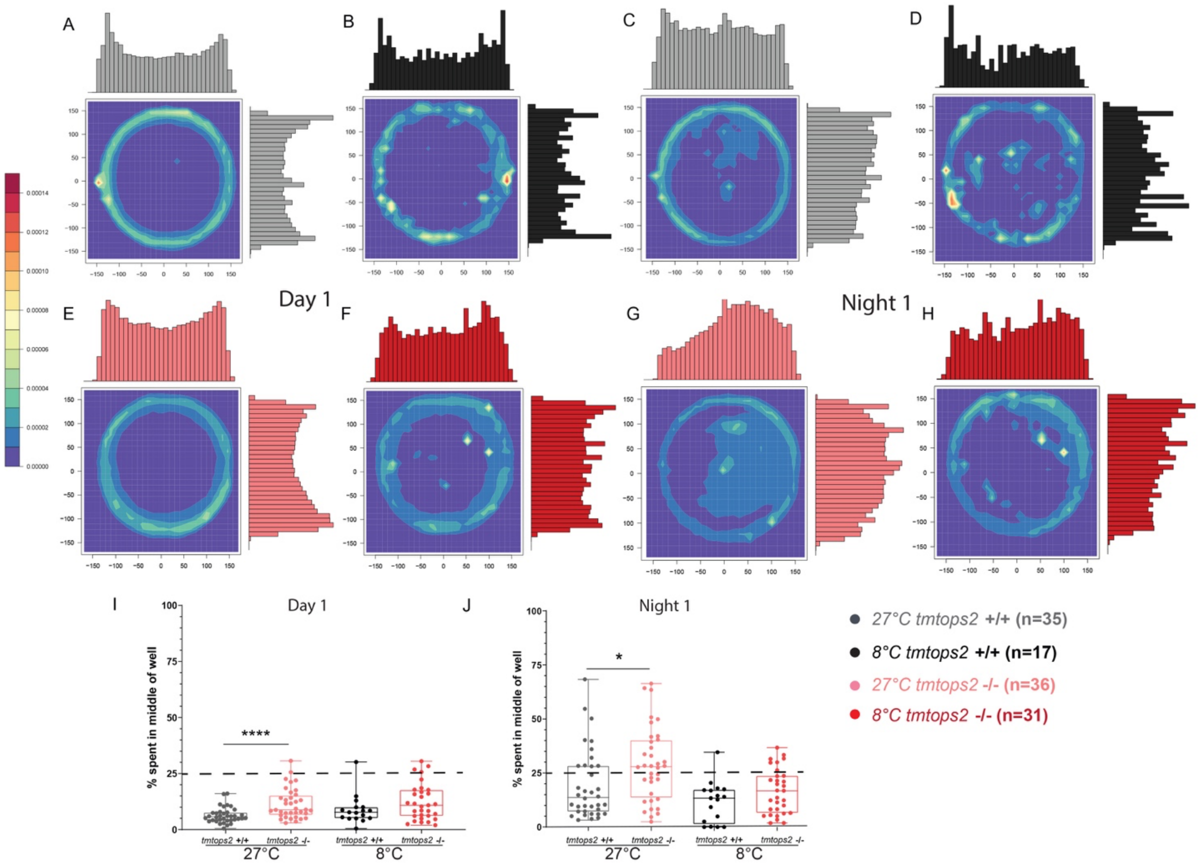

Supplementary Figure 2: refers to Figure 1I-K, 2F-H. (A-H) Heat plots and histograms showing density of the binned position datapoints for day 1 (A, B, E, F) and night 1 (C, D, G, H) at 27°C and 8°C, respectively. (I, J) Box plots showing the percentage of frames located in the middle zone, each dot represents one biological replicate i.e. one juvenile larva. **** $p \leq 0.0001$. * $p \leq 0.05$. a.u.: arbitrary units; dashed line represents expected % random threshold.

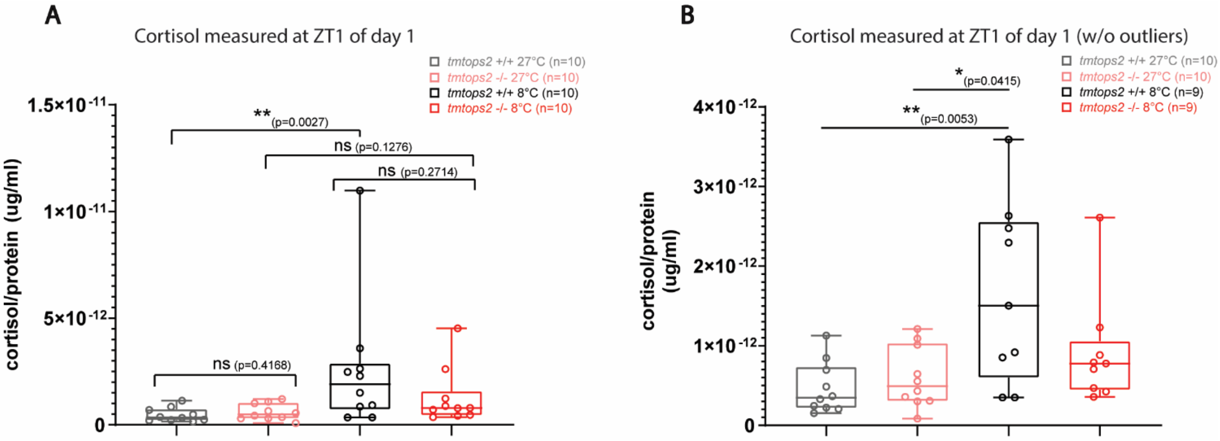

<u>Supplementary Figure 3:</u> Cortisol levels measured at ZT1 of the first day (depicted by the arrow in Fig.1A) as cortisol (µg/ml) over total protein levels (µg/ml) for wildtype and *tmtopsin2* mutant juvenile larvae at 27°C and 8°C. a.u.: arbitrary units. ** $p \leq 0.01$, * $p \leq 0.05$. ns = non-significant. (A) Statistics done for all animals (n = 10 per genotype and condition). (B) Statistical verification to test how much the statistical results depend on the two highest datapoints in the 8°C groups. Statistically significant differences seen in (A) remained significant, those that were non-significant remained non-significant.

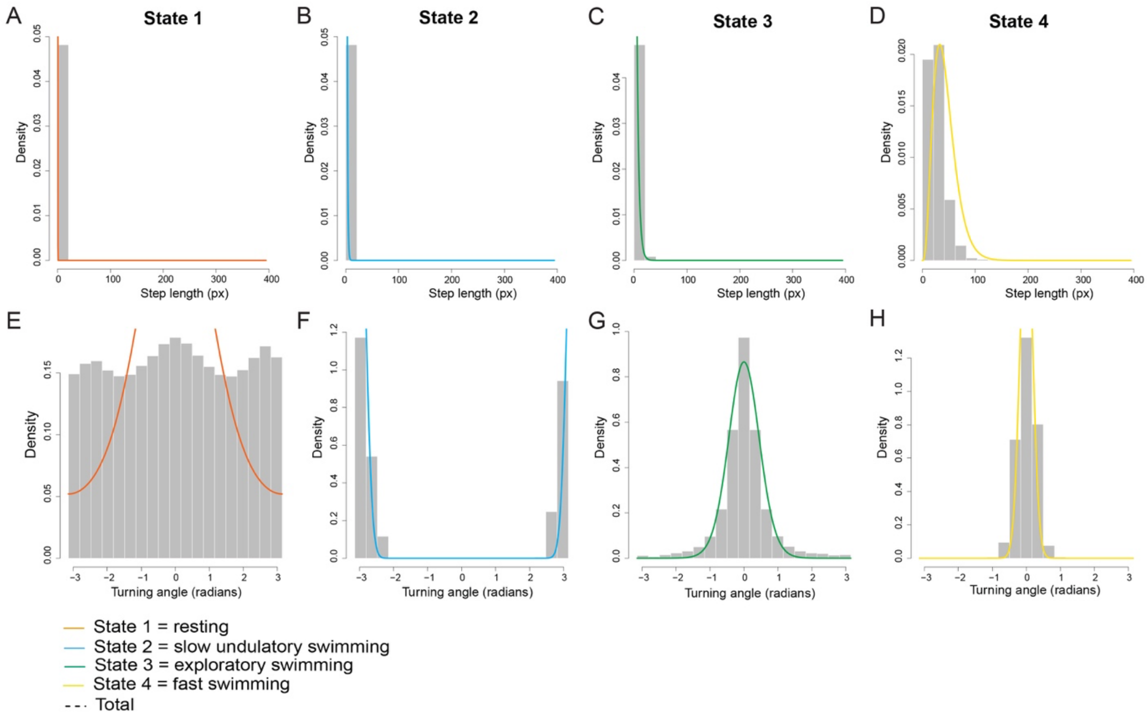

Supplementary Figure 4: refers to Figure 3. (A-D) Individual state curves for state 1 (A), state 2 (B), state 3 (C), state 4 (D) plotted over the step length histogram. (E, F, G, H) Individual state curves for state 1 (E), state 2 (F), state 3 (G), state 4 (H) plotted over the turning angle histogram.

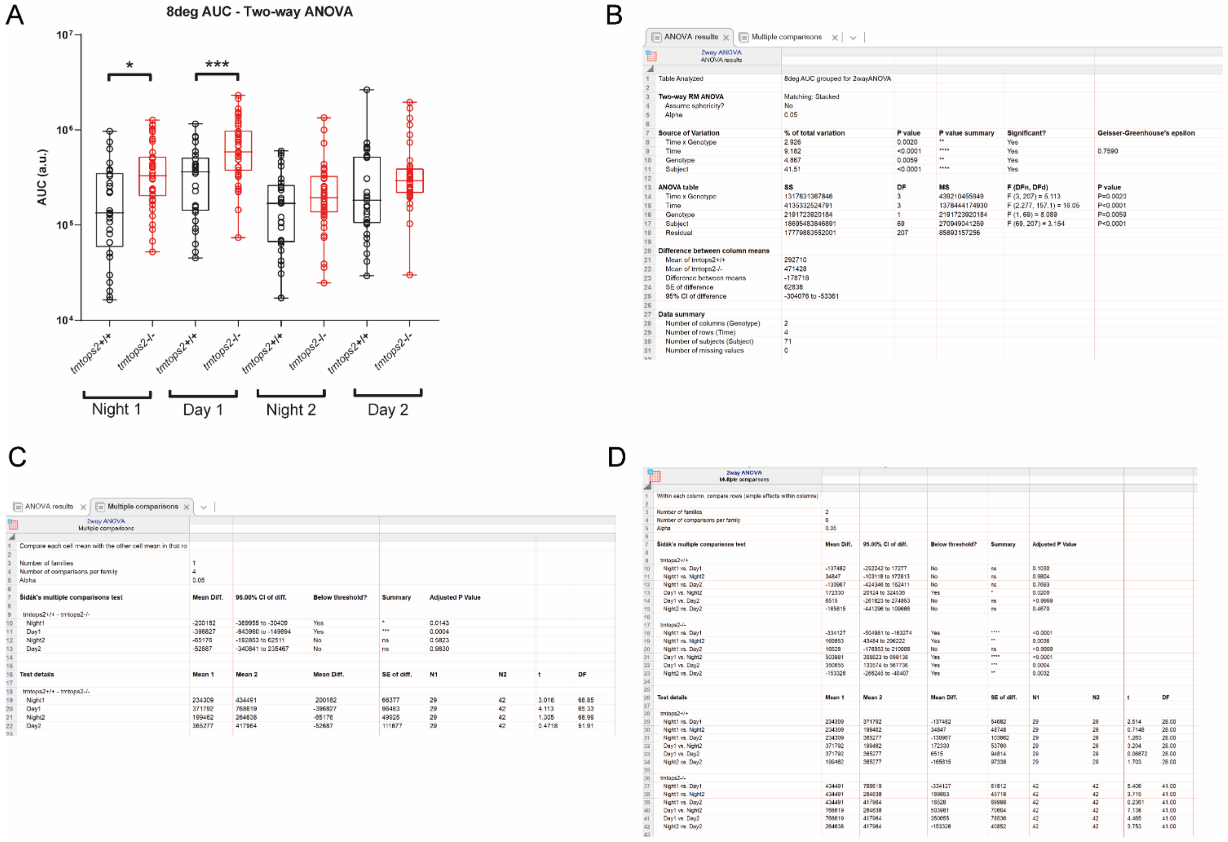

Supplementary Figure 5: Two-way repeated measures ANOVA with Geisser-Greenhouse correction followed by a Šidák's correction for multiple comparisons of the data shown in Figure 2 (B-E). * $p \leq 0.05$, ** $p \leq 0.01$, *** $p \leq 0.001$, **** $p \leq 0.0001$, ns = non-significant. (A) Locomotor activity (measured as AUC) during each separate night and day period. n = 29 (wildtype) and n = 42 (*tmt-opsin2*-/-). (B) Detailed results of two-way repeated measures ANOVA. (C) Detailed results of post-hoc multiple comparisons between genotypes. (D) Detailed results of post-hoc multiple comparisons between times.

Supplementary Movies- captions

<u>Supplementary Movie 1:</u> Representative 3-minute movie of a *tmt-opsin2* mutant juvenile medaka fish swimming in the 8°C environment.

<u>Supplementary Movie 2:</u> Representative 3-minute movie of a wildtype juvenile medaka fish swimming in the 8°C environment.

<u>Supplementary Movie 3:</u> Representative 3-minute movie of a wildtype juvenile medaka fish swimming in the 27°C environment.
